# Our Reprint

**Published:** 1842-12

**Authors:** 


*Our Reprint.*—Having on a former occasion expressed our opinion with regard to the character of the work, the republication of which, we commence in the library part of the present number of the Journal, it is unnecessary to say more concerning it. We do not think we could present our readers with a more acceptable treatise than this; and, it is our intention, as soon as the balance of the work shall be issued from the press in London, to republish it, also.

